# Reference values for the 6-min walking test in children and adolescents living in a moderate altitude region of Peru

**DOI:** 10.1186/s12887-023-04459-3

**Published:** 2024-02-27

**Authors:** Jose Sulla-Torres, Rubén Vidal-Espinoza, Christopher Avendaño-Llanque, Alexander Calla-Gamboa, Manuel Zúñiga-Carnero, Rossana Gomez-Campos, Marco Cossio-Bolaños

**Affiliations:** 1 Escuela de Ingeniería de Sistemas, Universidad Católica de Santa María, Arequipa, Perú; 2 Universidad Católica Silva Henriquez, Santiago, Chile; 3 Ciencias de La Actividad Física y del Deporte, Facultad de Ciencias de La Salud, Universidad San Ignacio de Loyola, Lima, Perú

**Keywords:** Reference values, Six-minute walk test, Children, Adolescents, Moderate altitude)

## Abstract

**Background:**

The assessment of cardiorespiratory fitness is important because it allows the identification of subgroups with poor health status and the targeting of effective intervention strategies to improve health.

**Objective:**

To compare the cardiorespiratory capacity of children and adolescents living in a moderate altitude region of Peru with international studies and to develop reference values for the 6-min walk test (6MWT) according to age and sex.

**Methodology:**

A descriptive cross-sectional study of schoolchildren from a region of moderate altitude in Peru was carried out. A total of 704 schoolchildren (400 males and 304 females) with an age range of 6 to 17 years were studied. Weight, standing height, waist circumference (WC), body mass index (BMI) and tri-ponderal mass index (TMI) were evaluated. The 6MWT was assessed in a straight line over a distance of 30 m. Percentiles were created through the LMS method [L (skewness: lambda), M (median: mu) and S (coefficient of variation: Mu)].

**Results:**

There were discrepancies in cardiorespiratory fitness performance with international studies by age and sex. The schoolchildren in the study reached stability and the highest number of meters in the last two age ranges (14 to 15 years: 698.1 m and 16 to 17 years 686.3 m in males). While females (14 to 15 years: 698.1 m and 16 to 17 years: 686.3 m). The proposed percentile values show ascending values as age advances. The cut-off points adopted are: low cardiorespiratory fitness < p25, moderate p25 to p75 and high cardiorespiratory fitness *p* > 75.

**Conclusion:**

We verified that the cardiorespiratory fitness evaluated by means of the 6MWT is ascending with the course of age. Even the performance with other countries is heterogeneous at early and middle ages, stabilizing during adolescence. The proposed reference values can be used to evaluate and monitor cardiorespiratory fitness during physical education classes.

## Background

Aerobic capacity is defined as the greatest amount of oxygen consumed during maximal exercise. It involves multiple systems, including the pulmonary, cardiac, vascular and musculoskeletal systems [1]. Studies indicate that they are strongly and inversely associated with individual cardiometabolic risk factors and metabolic syndrome in children and adults [2, 3]. Its assessment is performed through laboratory tests (gas analysis) [4] and indirectly through field tests [5].

Laboratory testing requires adequate infrastructure, sophisticated equipment and highly trained professionals. While field testing has emerged as an alternative to save time, resources and allow mass testing.

Indeed, in low and resource-poor settings, field and submaximal exercise testing should be implemented [6]. Especially if it is in developing countries and in public schools. Thus, aerobic fitness assessment in schools plays a key role in identifying students with declining levels [7].

For, in recent years, the importance of aerobic fitness in the school setting has been highlighted to promote optimal functioning and overall mental health wellbeing (e.g., depression, anxiety) as well as school performance [8, 9].

In this context, the 6-min walk test (6MWT) is an indirectly measured submaximal exercise test that allows quantification of functional exercise capacity in various clinical [10, 11] and epidemiological [12] populations.

Well, since the early 1970s it has increased its importance in clinical practice, epidemiology and research, due to its easy application, low cost and predictive ability in several cardiopulmonary disorders [13, 14].

It is currently considered an emerging method used to assess cardiorespiratory endurance in school children and adolescents applied in various geographical regions of the world [12, 15–18]. However, for a proper interpretation of the 6MWT test, appropriate reference values for the population of interest are necessary [19]. Percentiles in general can help to identify children and adolescents at risk for major chronic diseases not only by age and sex, but can also contribute to the evaluation of the effects of intervention programs developed in schools. For this purpose, it is relevant to have percentiles to monitor and quantify differences in performance between age and sex, respectively.

Consequently, given the differences in sociodemographic, morphological and physiological characteristics between studies [12, 17, 20, 21], it is necessary to develop reference values according to the sociocultural and demographic context for schoolchildren living in a moderate altitude region of Peru. Moreover, to our knowledge, no study to date has developed reference values in moderate altitude regions of Peru using the 6MWT.

It is widely known that the conditions and characteristics of moderate and high altitude geographic regions reflect low oxygen, low temperature, strong ultraviolet radiation and extreme climate fluctuations. Moreover, according to geo-referenced world population data, they have estimated that, in 2010, there were 83 million residents at > 2500 m [22] and, in 2017, 74.9 million lived at > 2500 m [23]. Thus, studying aerobic fitness in a moderate altitude region of Peru with marked geographical variations (coast, highlands and jungle) is highly relevant.

In fact, physical fitness monitoring is part of the physical education curricula and syllabi that emanate from the education ministries of countries in general. Such programs do not suggest the type of test to be applied in school systems. For example, in Peru, the Ministry of Education does not establish the use of a specific aerobic fitness test. Despite the fact that its population lives in varied geographical areas and is characterized as multicultural, and the only thing established in its guidelines is a clear and structured progression of achievement objectives covering all levels of schooling, highlighting the balanced development of the body and health [24].

Thus, the assessment of cardiorespiratory fitness by means of the 6MWT may serve to identify subgroups with poor health status and to guide effective intervention strategies to improve the health of the younger population [25].

This indirect test has demonstrated validity and reliability in school populations [26] and does not require maximum effort or expensive equipment and is endorsed by the American Thoracic Society [13].

Therefore, the objectives of the study were: a) to compare the cardiorespiratory fitness of children and adolescents living in a moderate altitude region of Peru with international studies and b) to develop reference values for the 6MWT according to age and sex.

## Methods

### Type of study and sample

A descriptive cross-sectional study was designed in children and adolescents from a moderate altitude region of Peru. The sample selection was non-probabilistic. We studied 704 schoolchildren (400 males and 304 females) with an age range of 6 to 17 years. The schoolchildren belonged to two state schools at primary level (6 to 11 years) and secondary level (12 to 17 years) living in the urban area of the city of Arequipa (Peru). This city is located south of Lima (Capital of Peru) and at 2320 m above sea level.

Both schools were informed about the objectives of the project. The principals of each school agreed to the study and informed the parents to sign the informed consent for their children. This procedure was explained to each parent (proxy) via email. The parents who accepted the acceptance sent the signed form. Then on the day of the evaluation, the children and adolescents signed the informed assent.

Schoolchildren from 6 to 17 years of age who did not have any type of physical walking limitation were included. Schoolchildren were excluded from the study if they could not perform any type of physical activity during school hours due to medical prescription.

The anthropometric measurements and the evaluation of the 6-min walking test were applied according to the suggestions described by the local ethics committee (UCSM-096–2022) and the Declaration of Helsinki (World Medical Medical Association) for human beings.

### Techniques and procedures

Anthropometric measurements were evaluated in the facilities of each school (physical education department). A team of 4 physical education professionals with extensive experience in anthropometric and physical fitness evaluation (6-min walk test) was formed.

The standardized protocol of Ross, Marfell-Jones [27] was used to evaluate anthropometric measurements (weight, height and CC waist circumference). Body weight (kg) was assessed using an electronic scale (Tanita BC 730 brand, UK) with a scale from 0 to 150 kg and with an accuracy of 100 g. Standing height was measured according to the Frankfurt plane using a portable stadiometer (Seca 216, Gmbh & Co. KG, Hamburg, Germany) accurate to 1 mm. The waist circumference (WC) was measured using a tape measure (Seca) to the nearest 1 mm. Body mass index (BMI) was calculated by the formula BMI: BMI = weight (kg)/height^2^ (m) and tri-ponderal mass index (TMI) by the formula: TMI = weight (kg)/height^3^ (m).

The evaluation of the 6-min walk test (6MWT) was performed according to the suggestions described by the American Thoracic Society ATS [13]. This test measures the distance a patient walks rapidly on a flat surface for a period of 6 min (6 MWT). It was performed in an open environment and on a flat surface 30 m long and 10 m wide. The surface was demarcated with colored adhesive tapes with a separation of two meters between the parallel lines (forming 5 lanes, one for each participant). The schoolchildren had to walk as many meters as possible during the six minutes in one direction back and forth. At the end of the six minutes, the distance covered by the participants (meters) was recorded. The test was carried out wearing sports clothing (T-shirt, shorts and sneakers).

Comparisons of cardiorespiratory fitness between the Peruvian study and international studies were made from two points of view: a) comparison of medians obtained in the 6MWT according to age and sex (schoolchildren from Croatia [18] and Italy [17]), b) comparisons of medians obtained in the 6MWT according to age range and sex (schoolchildren from Argentina [28] and Brazil [29]). The 4 international studies were performed at low altitude.

### Statistical analysis

The Kolmogorov–Smirnov (K-S) test was used to verify the normality of the 6-min walk test (6 MWT) data. Descriptive statistics (mean, standard deviation and confidence interval CI) were then calculated. Differences between both sexes were calculated by means of the t-test for independent samples. The LMS method [30] was used to propose percentiles. The LMS method uses the Box-Cox transformation to fit the data distribution to a normal distribution by minimizing the effects of skewness. The parameters L (skewness: lambda), M (median: mu) and S (coefficient of variation: Mu) were calculated according to the maximum penalized method [31]. Data processing was performed using LMS Chartmaker Pro software (The Institute of Child Health, London, UK) [32]. Comparisons of the 6-min walk test (6 MWT) with other studies were performed graphically using the 50th percentile (p50), according to age and sex. In all cases *p* < 0.05 was considered significant. Calculations were performed in Excel spreadsheets and SPSS 18.0.

## Results

The characteristics of the sample studied by age range and sex are shown in Table 1. In males there were significant differences between both age groups, for example, boys aged 12 to 17 years evidenced higher values of weight, height, BMI, WC, 6MWT (meters and km/h) (*p* < 0.000). However, in TMI, there were no differences between the two groups (*p* = 0.711). In females, there were significant differences in all the variables analyzed, where the group aged 12 to 17 years, showed higher values in anthropometric measurements, as well as in meters and km/h in the 6MWT than their similar groups aged 6 to 11 years.

**Table 1:** Anthropometric data and 6MWT values by age ranges and sex

Variables	**6 to 11 years**		**12 to 17 years**		**p**	**IC**	
	**X**	**SD**	**X**	**SD**		**Lower**	**Upper**
Males							
N	134		266				
Weight (kg)	34.3	11.3	58.2	11.7	0.000	-27	-20.7
Height (cm)	133.2	10.3	161.4	8.2	0.000	-30.6	-25.8
BMI (kg/m^2^)	18.9	4.1	22.3	3.8	0.000	-4.4	-2.2
TMI (kg/m^3^)	14	3.2	13.8	2.5	0.711	-0.6	0.9
WC (cm)	68.8	11.6	76.6	9.8	0.000	-10.9	-4.9
6MWT (m)	485.8	99.7	678.6	107.9	0.000	164.3	221.4
6MWT VM (m/h)	49000	1000	68000	1100	0.000	16000	22000
Females							
n	120		184				
Weight (kg)	33.5	11.3	55.1	10.6	0.000	-24.8	-18.4
Height (cm)	134.1	11.8	154.3	5.2	0.000	-22.6	-17.8
BMI (kg/m^2^)	18.5	4.0	23.1	4.0	0.000	-5.8	-3.3
TMI (kg/m^3^)	13.8	2.7	15	2.6	0.005	-2.0	-0.4
WC (cm)	67.1	11.2	72.9	9.2	0.000	-9.1	-2.7
6MWT (m)	467.5	75.9	641.8	106.3	0.000	147.5	201.1
6MWT VM (m/h)	47000	8000	64000	1100	0.000	15000	20000

*X* Mean, *SD* Standard deviation, *BMI* Body mass index, *TMI* Tri-ponderal mass index, *WC* Waist circumference, *MV* Mean velocity, *6MWT* 6-min walk test

**Fig. 1:**
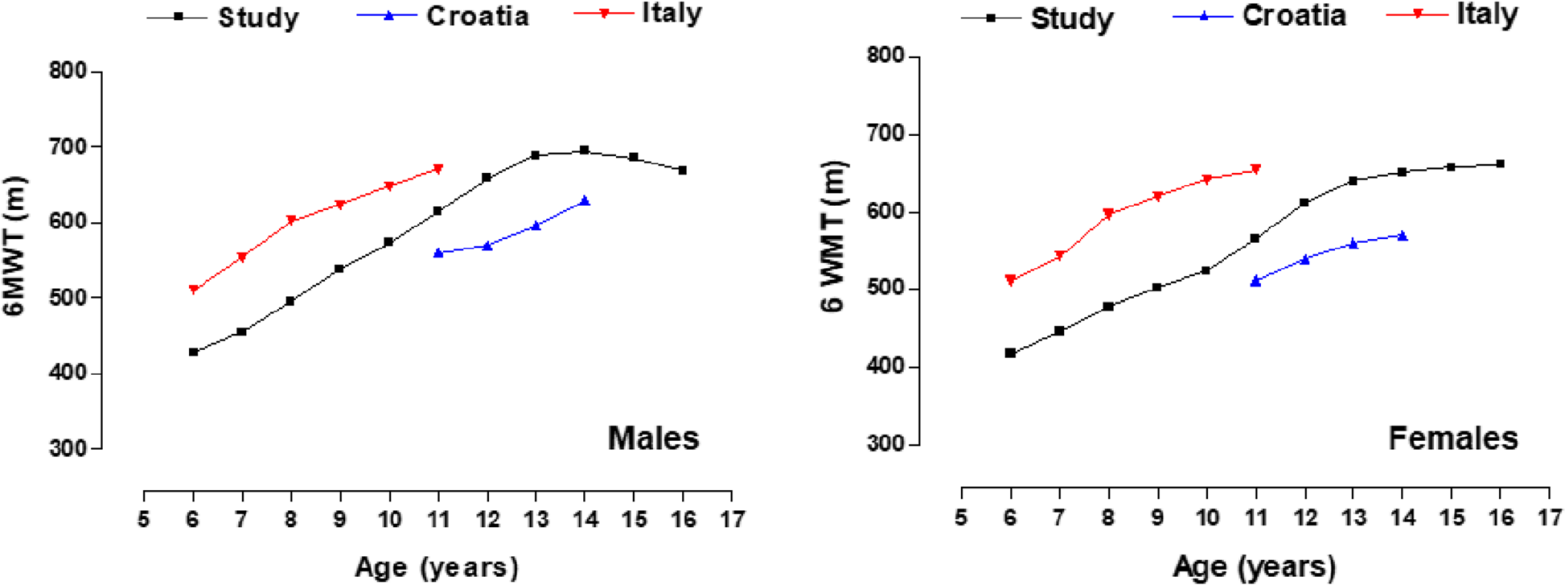
Comparison of medians (p50) of 6MWT by age and sex with international studies

Comparisons of the medians obtained in the 6MWT according to age and sex between schoolchildren in the study vs schoolchildren from Croatia [18] and Italy [17] are observed in Fig. 1. In all three studies the values are ascending as age increases. However, schoolchildren from Italy reflect better results in both sexes relative to schoolchildren from Arequipa (Peru), being higher from ∼55.7 to ∼107.2 m in males and from ∼88.9 to ∼118 m in females. In addition, schoolchildren from Arequipa (Peru) reflected better performance than their counterparts from Croatia in both sexes (e.g., in males from ∼55.1 to ∼88.0 m and in females from ∼54.1 to ∼80.8 m).

**Fig. 2:**
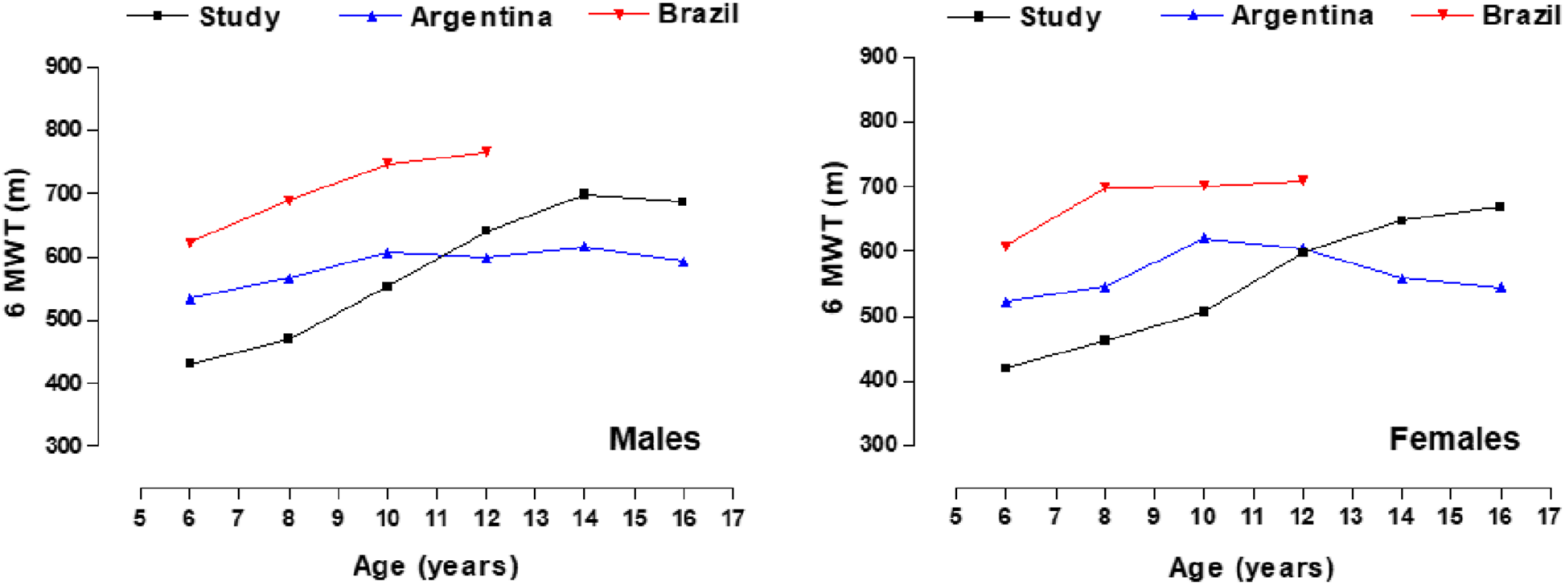
Comparison of medians (p50) of 6MWT by age range and sex with international studies

Comparisons of the medians obtained in the 6MWT according to age range and sex between the schoolchildren in the study vs. schoolchildren from Argentina [28] and Brazil [29] can be seen in Fig. 2. Note a clear performance superiority of Argentine and Brazilian schoolchildren over schoolchildren from Arequipa (Peru) at early ages, whose meters run shows better cardiorespiratory performance (e.g., in males from ∼53 to ∼228 m and in females from ∼6.3 to ∼240 m, respectively). In contrast, during adolescence, schoolchildren of both sexes from Arequipa (Peru) clearly outperformed their Argentine counterparts, showing a positive trend until 14–15 years of age, and then stabilizing at 16–17 years of age, respectively.

**Fig. 3:**
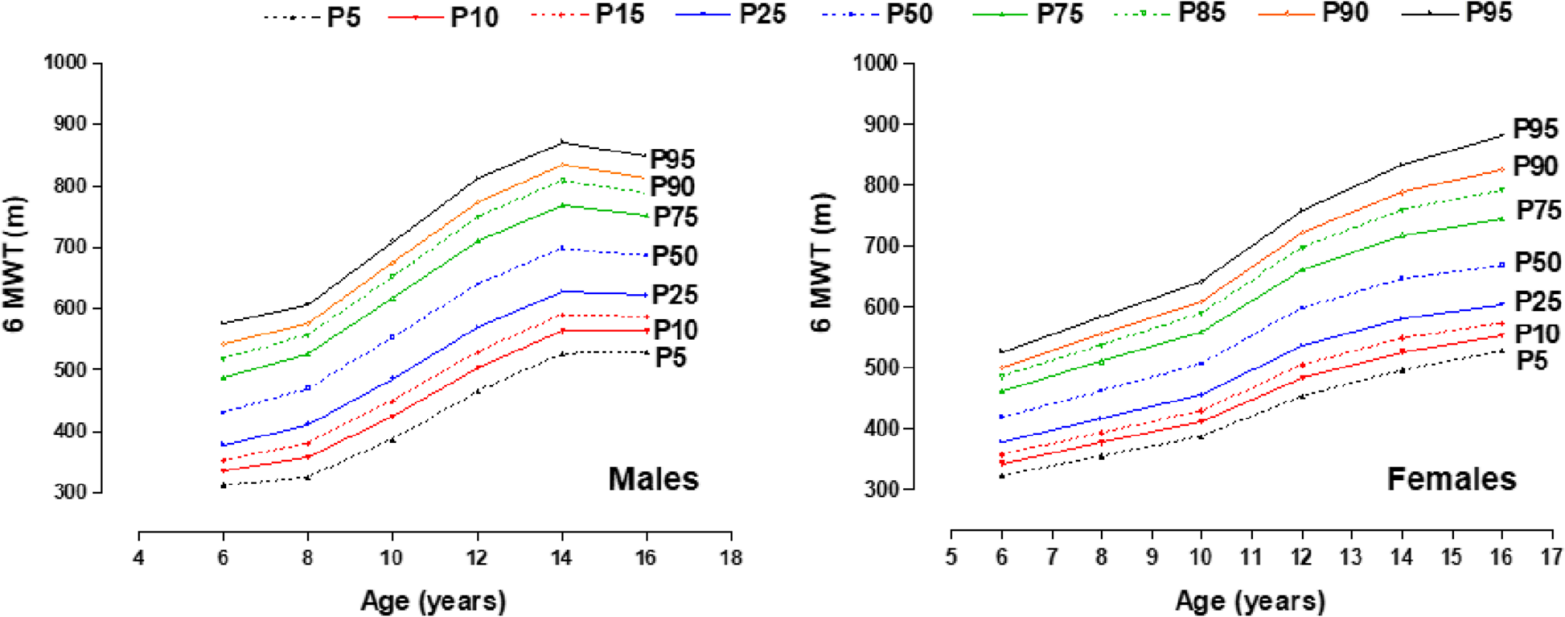
Reference for the 6MWT by age range and sex

The reference values for the 6MWT by age and sex are presented in Table 2. The test values show an upward trend as the age range progresses. At initial ages (6 to 7 and 8 to 9 years) the medians are relatively similar between both sexes. However, from 10 to 11 years of age, discrepancies begin to appear, where males present a better performance until 16 to 17 years of age. This superiority ranges from ∼18 to ∼51 m approximately. Figure 3 shows the percentile distribution graphs for both sexes.

**Table 2:** Reference values for the 6MWT (P3, P5, P10, P15, P25, P50, P75, P85, P90, P95, and P97) in children and adolescents of both sexes

Age	L	M	S	P3	P5	P10	P15	P25	P50	P75	P85	P90	P95	P97
Males														
6 a 7.99	0.31	430.9	0.19	298	313	337	353	379	431	487	519	542	577	600
8 a 9.99	1.16	470.0	0.18	305	326	359	381	412	470	527	557	577	607	626
10 a 11.99	1.20	552.7	0.18	362	387	425	450	486	553	618	652	675	709	731
12 a 13.99	1.05	639.8	0.16	440	465	504	530	569	640	711	749	774	812	837
14 a 15.99	0.93	698.1	0.15	503	527	564	590	628	698	769	808	834	872	897
16 a 17.99	0.85	686.3	0.14	509	530	564	587	622	686	752	788	812	848	872
Females														
6 a 7.99	0.54	419.4	0.15	311	323	344	358	379	419	462	486	502	527	543
8 a 9.99	0.43	462.8	0.15	342	356	378	394	417	463	511	538	557	585	604
10 a 11.99	0.48	506.7	0.15	372	388	413	430	456	507	560	590	610	642	662
12 a 13.99	0.55	598.2	0.16	434	454	484	505	537	598	663	698	723	760	785
14 a 15.99	0.14	646.9	0.16	478	497	527	549	581	647	719	760	789	834	864
16 a 17.99	-0.61	668.7	0.16	511	528	554	574	604	669	745	792	827	882	922

*P* Percentile, *L* Lambda, *M* Median, *S* Coefficient of variation

## Discussion

The initial objective of the study was to compare the cardiorespiratory capacity of children and adolescents living in a moderate altitude region of Peru with international studies. The results have evidenced discrepancies between the investigations, detecting differences in 6MWT when compared by age, age ranges and sex.

In fact, the schoolchildren in the study who live at moderate altitude in Peru have shown lower values in relation to their counterparts in other geographic regions, these differences are especially noticeable at early ages (six to 11 years). By then at advanced ages (12 to 17 years) adolescents of both sexes reached values similar to other studies [15, 33, 34].

However, a recent study conducted on children and adolescents residing above 3,500 m in Tibet showed lower aerobic fitness traits compared to their counterparts from lower altitude areas [35]. Apparently to the best of our knowledge, there is no consensus among studies, so there is a need to design research comparing aerobic fitness in schoolchildren living in three altitude regions. This information could help to clarify these inconsistencies.

In general, any differences in aerobic fitness between groups living in different altitude regions could be due to some factors that have to do with maturity status, level of energy expenditure, cultural differences and lifestyles between populations [35, 36].

In essence, the results obtained in this study are similar to other investigations with similar purposes [18, 37, 38]. Although due to the small sample size, sociodemographic characteristics and type of test used by the studies it is difficult to generalize the results obtained in this study. Therefore, more research is needed to identify the underlying reasons that may affect aerobic fitness in schoolchildren living in regions of moderate and high altitude.

These differences between studies and geographical regions may be due to several factors. Such as, for example, habitual walking speed or cultural aspects related to lifestyle, mood, attitude, subject and/or technician motivation when assessing 6MWT [39]. As well as weight status, sleep [40], cultural differences in physical education curricula, participation in sports [41], among other aspects.

In this sense, understanding the genetic, environmental and cultural contexts of normal development of children and adolescents is relevant [42]. Since this information can help professionals and specialists in the area to identify the 6MWT reference curve that best fits the particular needs of schoolchildren.

In this context, the second objective of this study was to develop reference values for the 6MWT according to age range and sex. In general, cardiorespiratory fitness reference values are widely used for functional assessment, prognosis and rehabilitation of pediatric and adult populations [43–45]. Thus, the percentiles proposed in the study can serve health professionals and physical education teachers as a fundamental tool for assessment in the school system.

Several studies have proposed percentiles for 6MWT [17, 29, 46, 47]. However, many of them do not cover large age ranges that complete childhood and adolescence. In that sense, the percentiles proposed here, cover a large age range (from 6–7 to 10–11 years, primary level and 12–13 years to 16–17 years, secondary level) as do some studies conducted in Argentina [28], Colombia [16] and Taiwan [48].

Indeed, the assessment of 6MWT among children and adolescents is important to identify subgroups with poor health status and to target effective intervention strategies to improve the health of the younger population [25]. Thus, the cut-off points adopted in this study were based on some recent research [16, 28], in which they suggest < p25 as low cardiorespiratory fitness, p25 to p75 as moderate and *p* > 75 high cardiorespiratory fitness.

Based on the above suggested, it is possible to evaluate the cardiorespiratory capacity of the studied children and adolescents. Therefore, it is possible to introduce in the school system, specifically in physical education classes to assess functional exercise capacity, as well as the detection of those individuals with higher or lower cardiorespiratory risk [18]. Whereby, it is possible to identify healthy and unhealthy individuals [49]. It can even serve for the elaboration of intervention strategies and to develop public policies that promote the development of children's health [40].

In sum, this study has some weaknesses that are described below. First, it has to do with the sample, we used non-probabilistic sample selection and the size is relatively small. So these aspects prevent generalization to other contexts and realities. Secondly, it was not possible to collect data to verify reliability (test and retest) and, thirdly, it was not possible to evaluate physiological parameters that would allow us to control more precisely the evaluation of the 6MWT. Therefore, future studies should include these relevant aspects and thus achieve greater representativeness and generalization of the results.

The study also presents some strengths, for example, as far as is known, it is the first study carried out at moderate altitude in Peru and South America. It also has some practical implications, since this information can serve as a baseline for future comparisons of secular trends. It can even serve as a valuable tool to be introduced in the Peruvian school system, whose calculations and interpretations can be easily and simply performed at the following link: www: http://reidebihu.net/t6minaqp.php.

## Conclusions

In conclusion, this study verified that the cardiorespiratory capacity evaluated by means of the 6MWT is ascending with the course of age. Even the performance with other countries is heterogeneous at early and middle ages, becoming homogeneous and stabilizing during adolescence. Furthermore, cardiorespiratory fitness benchmarks can serve as a valuable tool not only for physical education teachers, but also for coaches, trainers, and fitness instructors to promote physical health among schoolchildren. The results suggest their use and application in health and educational settings.

## Data Availability

The datasets supporting the conclusions of this research article are available by emailing the corresponding author.

## Abbreviations

MI: Body mass index
TMI: Tri-ponderal mass index
WC: Waist circumf6erence
MV: Mean velocity
6MWT: 6-Minute walk test
